# Identity Matters for Life Satisfaction: Sequential Mediation of Peer Relationships and Physical Health Perceptions and the Moderating Role of Household Characteristics Among Multicultural Adolescents

**DOI:** 10.1002/brb3.71317

**Published:** 2026-11-13

**Authors:** Yiran Li, Suyeon Lee

**Affiliations:** ^1^ BK21 Social Education Research Team, Department of Sociology, College of Liberal Arts Korea University Seoul Republic of Korea; ^2^ Department of Business Administration Chungnam National University Daejeon Republic of Korea

**Keywords:** health perception, household characteristics, identity, life satisfaction, peer relationships

## Abstract

**Background:**

This longitudinal secondary analysis examined the causal relationship between identity awareness and life satisfaction among multicultural adolescents in South Korea, focusing on the mediating role of peer relationships and physical health perceptions.

**Methods:**

Three waves of panel data from the Multicultural Adolescents Panel Study (*N* = 2271) were utilized to conduct autoregressive cross‐lagged modeling, covariance‐based structural equation modeling, and moderated mediation analysis.

**Results:**

Identity awareness at Time 1 significantly predicted life satisfaction at Time 3 through peer relationships and physical health perceptions at Time 2. The sequential mediation pathway, identity awareness → peer relationships → physical health perceptions → life satisfaction, exhibited significant indirect effects. The indirect effect of identity awareness on life satisfaction via physical health perceptions was stronger among adolescents with higher perceptions of family economic status than among those with lower perceptions of family economic status.

**Conclusions:**

This study contributes to a nuanced understanding of how identity interacts with interpersonal and contextual factors to shape multicultural adolescents’ well‐being.

## Abstract 2

Using three‐wave panel data from multicultural adolescents, we tested whether identity predicts life satisfaction through a sequential pathway from peer relationships to perceived physical health. The model supports the sequential indirect association and indicates that household characteristics moderate the strength of key links in the pathway.

## 1 Background

Globalization and international migration have increased cultural diversity worldwide, heightening concerns regarding social integration, especially among adolescents. In South Korea, the proportion of students from multicultural families rose for the 11th consecutive year in 2023, reaching 3.5% of total enrollment. This trend underscores the nation's transition from a traditionally homogeneous society to a multicultural one (KOSIS 2023; Statistics Korea 2023). Consequently, South Korean youth are increasingly exposed to culturally varied interactions (Arnett 2003). Against this backdrop, a key social challenge is the adaptation and well‐being of multicultural adolescents. Research on multicultural youth in South Korea has tended to emphasize potential challenges for them, such as depression, maladjustment, and social withdrawal (Jeong and Paik 2020; Kang et al. 2022), with less attention paid to protective mechanisms and positive developmental processes that foster their resilience and well‐being (Seligman and Csikszentmihalyi 2000; Kang 2019). Identity formation theory suggests that positive development depends on adolescents’ ability to balance diverse self‐concepts and social roles (Miller and Collette 2019). Achieving this balance is supported by positive exchanges with members of the cultural environment (Gardiner and Kosmitzki 2018) and fostering clarity in national identity, which is particularly important for multicultural adolescents (Arnett 2002). However, cultural clashes experienced during adolescence can disrupt this process, negatively affecting peer relationships, perceived health, and overall life satisfaction (Gardiner and Kosmitzki 2018; Miller and Collette 2019). Therefore, understanding how multicultural adolescents in South Korea perceive and negotiate their national identity alongside heritage and host‐society identities is pivotal in shaping their developmental trajectories and overall well‐being.

Cultural psychology further clarifies why positive identity formation is important in South Korea's collectivist context. Identity formation is closely related to self‐construal—how people make sense of their inner world—which varies significantly across cultural contexts. While Western contexts emphasize independent selves, many non‐Western contexts, such as South Korea's, emphasize interdependent selves oriented toward social harmony and the expectations of others (Markus and Kitayama 1991). Identity conflict can emerge when these self‐construals clash with each other. Furthermore, cultural identity is “negotiated” in social interactions, and conflicts can arise when individuals’ self‐perceptions diverge from how others categorize them. This misalignment between self‐identification and external categorization can trigger identity strain (Hong et al. 2016). In addition, identity conflict can result in anxiety, stress, social isolation, and diminished life satisfaction (Wiley and Deaux 2010; Kang 2019). Such negotiation dynamics are particularly salient for multicultural adolescents in South Korea, where the collective context emphasizes interdependence and achieving social harmony with others.

Contextual factors such as peer relationships and perceived health are also crucial for the well‐being of adolescents. Peer support can buffer stress and scaffold adaptive functioning, while health perceptions capture proximal appraisals linked to subjective well‐being. Family economic conditions—often conceptualized through the family stress perspective—may further shape or moderate these linkages by constraining resources and amplifying exposure to stress. Although peer support and perceived health are generally considered protective factors for well‐being, the strength of their influence can vary according to adolescents’ subjective perceptions of their family's socioeconomic status (i.e., subjective SES). According to the family stress model, economic pressure heightens parental emotional distress and parenting stress, strains family relationships, and ultimately, undermines adolescents’ psychosocial adjustment (Branje et al. 2009; Conger et al. 1992; Conger and Donnellan 2007; Masarik and Conger 2017). The stress‐buffering hypothesis of social support suggests that protective effects of peer support may be greater in low‐SES contexts; conversely, severe economic pressure may erode the benefits of support (Cohen and Wills 1985). The reserve capacity model likewise predicts that because low‐SES youth have fewer social–emotional resources, assets such as peer support should more strongly buffer stress–health pathways (Gallo and Matthews 2003; E. Chen and Miller 2013).

Subjective SES is also a robust predictor of adolescent health, independent of objective SES, and has been shown to moderate the associations among stress, social support, and well‐being (Goodman et al. 2001; Goodman et al. 2007; Quon and McGrath 2014). From a cumulative stress perspective, economic hardship increases exposure to chronic stress, increases stress reactivity, and weakens self‐regulation. Consequently, for youth from low‐SES families, the pathway linking peer support to life satisfaction through perceived health may be particularly consequential (Evans and Kim 2013). Accordingly, we hypothesize that subjective SES moderates the strength of this sequential pathway; we test this hypothesis by estimating interaction terms in a longitudinal model. Specifically, we use a three‐wave longitudinal design to examine how national identity relates to peer support, perceived health, and life satisfaction among multicultural adolescents in South Korea. We also test whether perceived family economic status moderates these associations. By integrating intrapersonal (i.e., identity) and contextual (i.e., peer, health, and socioeconomic) factors, this study addresses a significant deficit in Korean research on multicultural youth. The results of this study are expected to contribute to broader discussions on identity development, acculturation, and resilience by illuminating pathways to well‐being for an increasingly diverse youth population.

### 1.1 Link Between Identity and Life Satisfaction

Identity development is a critical task in adolescence (Erikson 1968), and its importance is amplified for multicultural adolescents who must navigate multiple cultural systems. A stable and coherent sense of identity provides the foundation for psychosocial health, positive adjustment, and life satisfaction (Gardiner and Kosmitzki 2018; Park 2023). Empirical studies in Korea further indicate that multicultural adolescents with strong identity awareness report higher levels of well‐being and adaptive functioning, whereas those with identity uncertainty or conflict are more likely to experience loneliness, psychological distress, and diminished life satisfaction (Cho and Jeong, 2024; Kim and Kim 2022; Yoon and Kwon 2025). Identity scholarship commonly distinguishes between identity content (i.e., the domains that define “who one is,” such as national, ethnic, personal, relational, or vocational identities) and identity processes (i.e., the exploration, commitment, and reconsideration through which identities are formed, maintained, and revised over time) (Crocetti 2017). In the MAPS, the available indicators capture adolescents’ emotional identification with and sense of belonging to Korean society. Accordingly, in this study we use the term identity awareness to denote the content/affective‐commitment component of national social identification rather than the full set of identity processes. We nevertheless draw on process‐oriented accounts by viewing national identification as a downstream outcome of ongoing cultural negotiation and identity work, which can subsequently function as a psychosocial resource that supports social integration and well‐being in everyday contexts.

Previous studies highlight the mechanisms through which identity awareness contributes to life satisfaction. Adolescents with a clear identity are better able to establish meaningful peer relationships, develop a sense of belonging, and integrate life experiences in ways that foster efficacy and coherence (Miller and Collette 2019; De Lise et al. 2024). In contrast, low identity awareness has been linked to school maladjustment, behavioral difficulties, and reduced well‐being (Shen and Kogan 2021; Mercer et al. 2017).

From the acculturation theory perspective (Berry 1997; Schwartz et al. 2010), multicultural adolescents who adopt integrative strategies, namely balancing heritage and host cultures, experience greater identity stability, psychological security, and life satisfaction (Crocetti et al. 2024; Mastrotheodoros et al. 2024; Karaś et al. 2014; Mok 2022). Conversely, identity conflict or ongoing reconsideration of identity commitment can undermine life satisfaction and adjustment (Crocetti et al. 2009; Hatano et al. 2016; Karaś and Cieciuch 2018).

These findings suggest that identity awareness is a positive predictor of life satisfaction among multicultural adolescents, while weak or unstable identity poses significant risks for reduced well‐being. This perspective provides the theoretical foundation for examining how identity awareness relates to life satisfaction in the present study.

### 1.2 Can Identity Awareness Facilitate Improvements in Peer Relationships, Perceived Health, and Overall Life Satisfaction?

As already mentioned, a coherent sense of identity is associated with enhanced life satisfaction. Conversely, physical or mental health difficulties can undermine this relationship (Romero et al. 2020). For adolescents who negotiate multiple cultural contexts, positive perceptions of self, health, and social participation are particularly important in reinforcing their confidence to explore and commit to meaningful identity alternatives. Thus, identity awareness should be understood not only as a marker of psychological adjustment but also as a critical determinant of adolescents’ physical, social, and emotional well‐being.

Empirical research has increasingly supported the significance of identity awareness for adolescents’ life satisfaction across multiple domains. Those who struggle to integrate heritage and host cultural identities often experience identity conflict, which limits their ability to build supportive peer relationships and maintain academic and personal functioning (Shen and Kogan 2021). By contrast, adolescents with strong identity awareness demonstrate a greater capacity to establish meaningful interpersonal bonds, sustain healthier lifestyles, and achieve higher levels of life satisfaction.

Nevertheless, existing research does not fully explain the processes by which adolescents successfully achieve identity awareness. Studies have disproportionately emphasized negative outcomes, such as identity conflict and maladjustment, while neglecting the protective and beneficial functions of identity awareness. Even more critically, research has rarely addressed how identity awareness interacts with adolescents’ peer relationships and health perceptions over time.

These gaps in the research highlight the urgent need for comprehensive, longitudinal analyses of identity awareness as a developmental resource. By examining the autoregressive and cross‐lagged effects of identity awareness on peer relationships, health perceptions, and life satisfaction, the present study moves beyond deficit‐oriented perspectives and demonstrates how identity awareness operates as a key mechanism of resilience among multicultural adolescents.(Motti‐Stefanidi 2015; Ryff 2014).

### 1.3 Experience of Adolescents With a Migrant Background

Understanding how identity awareness influences adolescents’ life satisfaction requires close examination of mediating processes. Although identity awareness is widely regarded as a key predictor of adolescent well‐being, its effects are not necessarily direct. Research suggests that interpersonal competence mediates the relationship between identity awareness and life satisfaction (M.‐O. Seo 2022), while peer relationships and health perceptions influence this association. In supportive social environments, identity awareness strengthens the protective functions of peer connections and positive health perceptions, thereby enhancing life satisfaction. In contrast, when identity awareness is weak or characterized by internal conflict, it can erode these relational and health‐related resources, ultimately diminishing life satisfaction (Aral et al. 2025).

The mechanisms of identity awareness must be understood within the broader sociocultural context of migration, which has increased the diversity of contemporary societies (Bagci and Rutland 2020). Adolescents from migrant backgrounds often navigate the challenge of reconciling divergent cultural norms and expectations while striving to construct a coherent sense of self (Nguyen and Benet‐Martínez 2013). Such challenges are particularly salient in South Korea, where institutional and social frameworks for multicultural integration remain relatively underdeveloped compared to many Western contexts. While some adolescents succeed in negotiating a dual identity that incorporates both heritage and host cultures (Gardiner and Kosmitzki 2018), others experience weak connections to either culture, leading to identity marginalization (Karataş et al. 2023). Identity struggles may be especially disruptive for those who migrate mid‐adolescence, since the interruption of identity formation processes exacerbates psychological vulnerability and increases the likelihood of lower life satisfaction (M.‐O. Seo 2022; Aral et al. 2025).

Based on Erikson's (1968) theory of identity development, studies have demonstrated that multicultural adolescents and those from ethnic minorities are more likely to encounter difficulties in achieving identity coherence across interpersonal, educational, and migratory contexts. These difficulties are closely associated with increased risks of psychological distress and maladjustment (Wiley and Deaux 2010; Yoon and Kwon 2025). Such findings emphasize the need to clarify the mediating roles of peer relationships and health perceptions in linking identity awareness with life satisfaction using a longitudinal approach to capture the causal and reciprocal dynamics underlying these associations.

In addition to these proximal processes, broader structural conditions should also be considered. Adolescents’ developmental trajectories are shaped not only by immediate interpersonal contexts but also by the socioeconomic resources and constraints of their families. Drawing on Bronfenbrenner's (2005) ecological systems theory, this study incorporates perceived family economic status (PFE) as a potential moderating factor. Empirical research indicates that perceived economic disadvantage amplifies psychosocial vulnerabilities by constraining access to supportive relational and health‐related resources, thereby increasing risks to well‐being (Reifsnider et al. 2005). Accordingly, the present study examines whether the indirect pathways from identity awareness to life satisfaction via peer relationships and health perceptions differ depending on adolescents’ PFE.

### 1.4 Current Study

The preceding theoretical background highlights significant gaps in the current literature concerning the mechanisms and contexts that shape the life satisfaction of multicultural adolescents. While prior research has emphasized the importance of identity awareness, peer relationships, and health perceptions, few studies have examined how these factors interact within a multidimensional framework of well‐being or considered the complex influences of migration background and family economic status.

In response, the present study examines life satisfaction among adolescents by incorporating its cognitive, emotional, physical, and social dimensions based on contemporary models of subjective well‐being and eudaimonic flourishing (Diener et al. 1999; Keyes 2005; Ryan and Deci 2001; Ryff 1989). By situating these domains within the lived experiences of multicultural adolescents, this study provides a comprehensive foundation for exploring both psychological and contextual mechanisms underlying their well‐being.

Despite recognizing the multiple dimensions of life satisfaction, the study employs a single global construct to maintain analytic clarity and parsimony in longitudinal analyses. This choice aligns with evidence that, in Korean multicultural samples, well‐being items load primarily on a robust general life‐satisfaction factor rather than on distinct subdomains (Moon et al. 2018).

This study builds on, and should be read alongside, a previously published study by the research team that used the same participant cohort and dataset, namely Waves 1 to 3 of the second cycle of the MAPS (2019–2021; *N* = 2271) (Li et al. 2026). The two studies share the measures of national identity (termed identity awareness in the present study), perceived physical health, and life satisfaction; the same set of baseline covariates; and a similar analytic family of autoregressive cross‐lagged and longitudinal mediation models estimated with covariance‐based and partial least squares structural equation modeling. Both studies also examined the indirect path from identity to life satisfaction through perceived health. The earlier study, grounded in social identity theory, Berry's 2003 bidimensional acculturation model, and affective events theory, asked whether national identity promotes life satisfaction through bicultural acceptance attitudes and perceived health, and it estimated these indirect effects unconditionally, that is, as a single effect for all adolescents.

The present study departs from the earlier study in three respects. First, it examines a different mechanism. It asks whether identity awareness operates through peer relationships, measured with a peer relationship scale that was not used in the earlier study (which included only the number of close friends as a covariate), and it tests a sequential pathway from identity awareness through peer relationships to physical health perceptions and life satisfaction. Bicultural acceptance attitudes, the focal mediator of the earlier study, are not examined here. Second, it asks for whom the indirect pathways hold. It tests whether adolescents’ perceived family economic status (PFE) moderates the indirect effects by means of a conditional process (moderated mediation) analysis, whereas the earlier study examined no moderator. Third, it grounds these questions in the family stress model, the reserve‐capacity model, and ecological systems theory, frameworks that were not used in the earlier study. Because of the overlaps described above, findings for the shared constructs and for the shared health pathway are not independent of those reported in Li et al. (2026); the distinct contribution of the present study lies in the peer relationship pathway and in its conditioning by PFE. The relationship between the two sets of findings is considered further in Section 4.

Guided by the framework described above, this study investigates how identity influences overall life satisfaction, with a particular focus on the mediating roles of peer relationships (operationalized as perceived peer support) and health perceptions (operationalized as perceived health). Given the unique challenges associated with adolescents’ migration backgrounds, the study explores PFE as a potential moderator of these associations. By “developmental pathways,” we refer to the sequential process whereby peer support fosters perceived health, which in turn enhances life satisfaction (peer support → perceived health → life satisfaction). The analysis uses three annual waves of panel data (2019–2021); T1 denotes the baseline assessment, and T2 and T3 are follow‐ups conducted approximately 1 year and 2 years after baseline, respectively. We test the following hypotheses.

**Hypothesis 1**. Identity awareness at time (T) 1 positively predicts life satisfaction at T3.
**Hypothesis 2**. The relationship between identity awareness at T1 and life satisfaction at T3 is sequentially mediated by peer relationships and physical health perceptions at T2.
**Hypothesis 3**. Peer relationships at T2 mediate the relationship between identity awareness at T1 and physical health perceptions at T3.
**Hypothesis 4**. Physical health perceptions at T2 mediate the relationship between identity awareness at T1 and life satisfaction at T3.
**Hypothesis 5**. The indirect effects of identity awareness on life satisfaction via peer relationships and physical health perceptions are moderated by PFE; higher PFE is associated with stronger mediation effects.
**Hypothesis 6**. Identity awareness, peer relationships, physical health, and life satisfaction demonstrate autoregressive continuity across the three time points.

## 2 Methods

### 2.1 Participants

The participants were multicultural adolescents from the Multicultural Adolescents Panel Study (MAPS). The sample comprised 2271 students enrolled in upper elementary (Grade 4 and higher) and middle schools (up to Grade 9) across various regions in South Korea. The final analysis included only multicultural adolescents up to and including sixth grade in 2021. The panel was constructed by recruiting adolescents who, as of 2019, were enrolled in the fourth grade of elementary school. In this study, the term “multicultural adolescents” follows the Ministry of Education's definition of multicultural students and broadly includes children from international marriages, newly immigrated adolescents, and foreign‐national children. Children from international marriages are those born in Korea to a Korean parent and a foreign parent; newly immigrated adolescents are children of such couples who were born abroad, spent part of their childhood there, and subsequently entered Korea; and foreign‐national children are those born to two foreign parents. This definition is drawn from the Ministry of Education and the Korea Educational Development Institute (2018).

We explicitly disclose that the analytic sample is the same participant cohort and dataset analyzed in a previously published study by the research team (Li et al. 2026), which likewise used Waves 1 to 3 of the second cycle of the MAPS (2019–2021; *N* = 2271). The measures of identity awareness (national identity), perceived physical health, and life satisfaction described below, and the baseline covariates (sex, age, number of close friends, current academic concerns, and educational aspiration), are the same as those used in that study. The peer relationship scale and the indicator of PFE were not used in that study and are specific to the present analysis.

Participants’ average age was 10.13 years (SD = 0.44), with 1156 boys (50.9%) and 1115 girls (49.1%). Regarding residential area, 12.1% of participants resided in Seoul, 28.2% in Gyeonggi/Incheon metropolitan area, 17.5% in Chungcheong and Gangwon, 24.8% in Gyeongsang, and 17.4% in Jeolla and Jeju. In total, 33.7% of the participants lived in metropolitan areas, 51.6% in mid‐sized or small cities, and 14.7% in towns or rural areas.

Participants’ mothers’ ages ranged 24–59 years, with most being 37 (8.0%) and 33 years (7.4%). Fathers’ ages ranged 26–76 years, with most being 49 (7.1%) and 50 years (6.9%). The largest proportion of parents had a high school or middle school education or less (mothers: 44.5% and 29.5%, respectively; fathers: 53.0% and 11.8%, respectively); 15.2% of mothers and 17.1% of fathers held at least a bachelor's degree. The largest proportions of mothers were unemployed or other (29.8%) or machine operators or assemblers (14.7%), whereas the largest proportions of fathers were machine operators or assemblers (18.5%) or unskilled workers (15.1%). Most mothers were Vietnamese (33.6%) or Han Chinese and other Chinese ethnicities (17.3%), whereas most fathers were South Korean (73.1%) or Korean‐Chinese (7.2%). Most parents were married (89.6%) and some were divorced (7.4%).

### 2.2 Procedure

This study used the second wave of the MAPS, a survey designed to track the developmental trajectories of adolescents from multicultural families residing in South Korea, conducted by the National Youth Policy Institute (NYPI) of South Korea. The study was launched in 2019 with a baseline sample of fourth‐grade elementary school students and their parents, with annual follow‐up data collection until 2024, when the adolescents reached the third year of middle school. In total, six data collection waves (Waves 1–6) were planned. During each wave, the adolescents and either their mother or father completed separate self‐administered questionnaires. The dataset was fully anonymized before being made available to researchers, ensuring anonymity. The MAPS was approved by the Institutional Review Board of the NYPI in 2019 and 2020. All participants were informed about the study's purpose, procedures, and their right to withdraw, and provided voluntary consent to participate.

This study used de‐identified secondary data from the MAPS conducted by the NYPI, Republic of Korea. Ethical review and informed consent were completed as part of the original MAPS data collection. Although this study did not directly collect personal information or contact participants, an ethics determination (approval/exemption) was obtained from the researchers’ affiliated university's Institutional Review Board (approval numbers: 201904‐HR‐Unique‐002‐01; 202009‐HR‐Unique‐011). As the present study involved secondary analyses of anonymized data and no new data collection from human participants, additional institutional review board approval beyond this determination was not required.

T1, T2, and T3 referred to three consecutive assessment points derived from the second cycle of the MAPS (MAPS 2), as follows: T1 corresponded to Wave 1 (conducted in 2019; participants were in Grade 4), T2 to Wave 2 (2020, Grade 5), and T3 to Wave 3 (2021, Grade 6). Despite being named MAPS 2, the follow‐up assessments all followed the same cohort; therefore, T1–T3 represent the developmental trajectory of this cohort throughout their upper elementary school years.

### 2.3 Measures

#### 2.3.1 Demographics

Sociodemographic and background variables were systematically obtained at baseline (Wave 1). The dataset encompassed participants’ age, biological sex, parental educational attainment, and country of origin. Age was documented as a continuous variable in years. Biological sex was self‐reported and subsequently dummy‐coded for statistical processing (0 = male, 1 = female). Parental educational attainment was independently assessed for both mothers and fathers using predefined response categories (1 = middle school or less, 2 = high school, 3 = two‐year college/vocational college, 4 = four‐year university [bachelor's degree], 5 = graduate degree or higher). For analytical purposes, the highest educational level attained by either parent was utilized. Country of origin was classified according to the foreign‐born parent's country (primarily the mother, as indicated in the survey) and coded using the original categorical schema; when necessary, country categories were consolidated and represented as dummy variables in analyses.

Baseline covariates were also measured during Wave 1 and encoded according to initial response protocols. The number of close friends was operationalized as an ordinal variable reflecting the reported count (e.g., 0, 1–2, 3–4, 5 or more). Current academic concerns were assessed via a Likert‐type scale indicating the extent of perceived school‐ or grade‐related stress (*1 = not at all, 5 = very much*). Parental educational aspiration was measured by the highest level of education parents desired for their child, treated as an ordinal categorical variable in subsequent analyses.

#### 2.3.2 Identity Formation Among Multicultural Adolescents (Korean Social Identification)

During the second wave of the MAPS, identity was measured with four items adapted from the Korean Social Identification Scale's group involvement subscale, originally developed by Seong (2001). The items were revised for adolescents and assessed emotional identification with and a sense of belonging to South Korean society. Responses were rated on a 5‐point Likert scale (*1 = not at all true, 5 = very true*). A sample item was “When someone compliments Korea, I feel as if I am being praised.” In Seong's (2001) study, Cronbach's *α* for the subscale was 0.78. In the present study, Cronbach's *α* indicated good internal consistency across waves (T1 = 0.825, T2 = 0.840, T3 = 0.847). Composite reliability (CR) was consistently high (T1 = 0.884, T2 = 0.893, T3 = 0.897). Average variance extracted (AVE) supported convergent validity (T1 = 0.656, T2 = 0.676, T3 = 0.685).

#### 2.3.3 Life Satisfaction

Life satisfaction was assessed with three self‐report items from the Youth Development Index Survey, developed by S. Y. Kim et al. (2006) and revised by J. K. Kim et al. (2010). Items were rated on a 5‐point Likert scale (*1 = not at all true, 5 = very true*). A sample item is “I enjoy living my life.” In S. Y. Kim et al. (2006) study, Cronbach's *α* for the scale was 0.75. In the present study, Cronbach's *α* was stable across waves (T1 = 0.813, T2 = 0.807, T3 = 0.810). CR remained high and consistent (T1 = 0.889, T2 = 0.887, T3 = 0.888). AVE values were strong across waves (T1 = 0.729, T2 = 0.724, T3 = 0.727).

#### 2.3.4 Peer Relationships

Peer relationships were assessed using items from the social relationship domain of Kim's (2013) School Adjustment Scale, items from Hwang and Kim's (2006) Peer Relationship Scale, and one item developed by the research team, resulting in a four‐item scale. This scale was designed to assess adolescents’ social adjustment in peer relationships at school, based on perceived emotional intimacy, support, understanding, and respect. The items were adapted to reflect adolescents’ developmental level. The scale was first implemented in the seventh wave of the MAPS. A sample item is “My school friends understand me well.” All items were rated on a 5‐point Likert scale (*1 = not at all true, 5 = very true*). Peer relationships were measured with three items, and internal consistency reliability was relatively low in the present sample (Cronbach's *α* = 0.566). In the present study, Cronbach's *α* showed good reliability (T1 = 0.851, T2 = 0.844, T3 = 0.862). CR was uniformly high (T1 = 0.910, T2 = 0.906, T3 = 0.916). AVE also indicated strong convergent validity (T1 = 0.771, T2 = 0.762, T3 = 0.784).

#### 2.3.5 Psychological and Physical Health Scale

Health was assessed using 12 items adapted from Cawte (1972) and Hwang (1997) and modified for adolescents. The scale was divided into two subdomains: physical health (six items, selected from the eight in the original scale) and psychological health (six items, four from the original scale and two developed by the research team). The items covered common physical symptoms, emotional stability, stress responses, and mood states, and were rated on a 5‐point Likert scale (*1 = not at all true, 5 = very true*). In the present analyses, we used the physical health subscale (2 items) to operationalize perceptions of physical health; higher scores indicate more positive perceived physical health. In the present study, Cronbach's *α* was acceptable across waves (T1 = 0.805, T2 = 0.802, T3 = 0.793). CR remained high (T1 = 0.911, T2 = 0.910, T3 = 0.906). AVE values were consistently strong (T1 = 0.837, T2 = 0.834, T3 = 0.829).

#### 2.3.6 Perceived Family Economic Status

PFE was assessed with a single‐item indicator developed by the MAPS research team, asking adolescents to rate their household's economic condition using ordered response categories (e.g., from “very poor” to “very affluent”). This measure reflects perceived economic standing (subjective socioeconomic status) rather than objective household income; therefore, internal consistency indices (e.g., Cronbach's alpha or omega) are not applicable. Accordingly, we interpret PFE as adolescents’ subjective appraisal of household resources and living conditions, which may differ from objective socioeconomic indicators. Accordingly, PFE was treated as a subjective SES indicator, and our interpretations are limited to perceived household standing rather than objective socioeconomic conditions.

### 2.4 Data Analysis

Descriptive statistical analyses were performed using SPSS Statistics for Windows (version 29.0; IBM). All subsequent analyses were conducted using SmartPLS 4.1 (Hair et al. 2024), employing partial least squares (PLS) and maximum likelihood robust estimators (Kline 2023). To examine longitudinal relationships between identity awareness, peer relationships, physical health perceptions, and life satisfaction, covariance‐based structural equation modeling (CB‐SEM) was used. Amos version 29.0 was used to conduct longitudinal measurement invariance testing, autoregressive cross‐lagged panel modeling, and mediation analyses to assess temporal stability and causal pathways. CB‐SEM was chosen because it enables the evaluation of construct validity and measurement invariance over time, providing stable estimates for cross‐lagged paths and indirect effects among latent variables.

#### 2.4.1 Conditional Process Analysis (PROCESS in SmartPLS)

To test whether the indirect effect of identity awareness on life satisfaction through physical health perceptions varied by PFE, a moderated mediation analysis was performed using the PROCESS macro for SmartPLS based on PLS‐SEM (Hayes 2018). The inclusion of latent variable interactions in CB‐SEM can increase statistical complexity and cause interpretive instability (Hayes et al. 2017). Assumption violations can result in estimation errors, particularly in conditional process models involving multiple interactions, as used in this study (Cham et al. 2017). Considering these limitations, a PROCESS macro‐based PLS‐SEM approach was adopted to provide a more stable estimation of conditional indirect effects with moderator variables. Although this method has some limitations in addressing measurement error, its estimation accuracy is comparable to CB‐SEM (Hayes 2018; Ratzmann et al. 2016), making it a practical and efficient tool. Prior to hypothesis testing, longitudinal measurement invariance was assessed for each construct. Metric invariance was established for all latent variables, supporting the validity of the measurement model and justifying its use in hypothesis testing.

## 3 Results

### 3.1 Cross‐Lagged Associations of Identity Awareness and Life Satisfaction

#### 3.1.1 Cross‐Lagged Associations among Identity Awareness, Peer Relationships, Physical Health Perceptions, and Life Satisfaction

An autoregressive cross‐lagged panel model was specified to examine reciprocal longitudinal associations among national identity awareness, peer relationships, physical health perceptions, and life satisfaction across three waves. Each model adjusted for the following conditions: (a) temporal stability or autoregressive paths (T1→T2→T3); (b) within‐time correlations between variables (e.g., correlations at T1 and correlated changes at T2 and T3); (c) the participant‐level covariates: sex (0 = male, 1 = female), age, number of close friends, educational aspiration level, and current academic or school‐related concerns (e.g., studying, grades, school life, entrance exams, or career path).

To derive the most parsimonious model of reciprocal associations, the initial model (M1) was specified with all paths freely estimated. Following Kline (2023), model fit was assessed using a combination of fit indices: comparative fit index (CFI) values > 0.90 and Tucker–Lewis Index (TLI) values > 0.95 were considered indicative of acceptable and excellent fit, respectively, and the same was true for root mean square error of approximation (RMSEA) and standardized root mean square residual (SRMR) values < 0.08 and < 0.05, respectively. An upper bound of the 90% confidence interval for an RMSEA < 0.10 was considered evidence of acceptable model fit (Kline 2023).

Longitudinal measurement invariance tests were conducted for identity awareness, peer relationships, physical health perceptions, and life satisfaction. The results indicated that the range of chi‐square minimum (CMIN)/df values was 2.155–2.470, and changes in CFI (*Δ*CFI = 0.977–0.982) and TLI (*Δ*TLI = 0.972–0.978) exceeded the recommended thresholds, confirming that the measurement model functioned consistently across time points. Therefore, longitudinal measurement invariance was established, justifying the subsequent structural model analyses.

Subsequently, the models were sequentially tested by imposing equality constraints on (a) cross‐lagged paths across T1→T2→T3, (b) cross‐lagged paths and within‐time correlations across time points, and (c) error variances and covariances across time points. These models were compared regarding their weak, strong, and strict invariances. Based on three commonly used criteria (i.e., significant *Δχ* ^2^ at *p* < .05, ΔCFI ≥ −0.010, and ΔRMSEA ≥ 0.015), strict measurement invariance was confirmed if at least two of these criteria were satisfied (F. F. Chen 2007).

The results indicated that all four models, measurement invariance, autoregressive invariance, cross‐lagged invariance, and residual/covariance invariance, met the criteria for time invariance across the specified parameters. Thus, the strict invariance model was retained as the final model. All constructs exhibited high temporal stability.

All models included sex and age as covariates without parameter constraints, allowing their effects to vary over time. Furthermore, multigroup analyses were conducted to determine whether significant differences in path coefficients and correlations existed between adolescents from the majority group and those with a migrant background (Table 1).

**TABLE 1:** Correlations and within‐time correlations across measurement waves.

Period	*M*	SD	T1(A)	T1(B)	T1(C)	T1(D)	T2(A)	T2(B)	T2(C)	T2(D)	T3(A)	T3(B)	T3(C)	T3(D)
**T1(A)**	2.623	0.717	1											
**T1(B)**	3.850	0.816	0.230^**^	1										
**T1(C)**	3.491	0.548	0.160^**^	0.344^**^	1									
**T1(D)**	3.345	0.556	0.211^**^	0.400^**^	0.469^**^	1								
**T2(A)**	2.679	0.704	0.358^**^	0.179^**^	0.144^**^	0.131^**^	1							
**T2(B)**	3.798	0.704	0.135^**^	0.413^**^	0.252^**^	0.264^**^	0.249^**^	1						
**T2(C)**	3.481	0.533	0.118^**^	0.236^**^	0.364^**^	0.289^**^	0.162^**^	0.319^**^	1					
**T2(D)**	3.251	0.549	0.127^**^	0.223^**^	0.258^**^	0.391^**^	0.197^**^	0.319^**^	0.415^**^	1				
**T3(A)**	2.788	0.669	0.262^**^	0.116^**^	0.128^**^	0.114^**^	0.435^**^	0.162^**^	0.130^**^	0.153^**^	1			
**T3(B)**	3.855	0.683	0.130^**^	0.333^**^	0.190^**^	0.238^**^	0.158^**^	0.463^**^	0.258^**^	0.265^**^	0.207^**^	1		
**T3(C)**	3.452	0.558	0.117^**^	0.198^**^	0.286^**^	0.265^**^	0.170^**^	0.242^**^	0.435^**^	0.308^**^	0.191^**^	0.299^**^	1	
**T3(D)**	3.214	0.544	0.113^**^	0.161^**^	0.183^**^	0.295^**^	0.123^**^	0.225^**^	0.265^**^	0.410^**^	0.202^**^	0.327^**^	0.501^**^	1

*Note*: T1 = Time 1, T2 = Time 2, T3 = Time 3; (A) Identity awareness; (B) Peer relationships; (C) Physical health perceptions; (D) Life satisfaction.

^***^ *p* < 0.001.

#### 3.1.2 Autoregressive Cross‐Lagged Effects of Identity Awareness, Peer Relationships, Physical Health Perceptions, and Life Satisfaction

The model used for analyzing cross‐lagged effects demonstrated good fit (*χ* ^2^ = 1102.322, df = 520; CMIN/df = 2.120, goodness‐of‐fit index (GFI) = 0.974, adjusted goodness‐of‐fit index (AGFI) = 0.966, CFI = 0.985, normed fit index (NFI) = 0.972, incremental fit index (IFI) = 0.985, TLI = 0.982, RMSEA = 0.022, SRMR = 0.043), with most indicators suggesting excellent model fit (Byrne 2012; Kline 2023).

All autoregressive paths were statistically significant: identity awareness at T1 → T2 → T3 (*β* = 0.200, SD = 0.017, *t* = 11.910, *p* < 0.001; 95% confidence interval [CI] [0.168, 0.233]); physical health at T1 → T2 → T3 (*β* = 0.139, SD = 0.016, *t =* 8.476, *p* < 0.001; 95% CI [0.107, 0.172]); peer relationships at T1 → T2 → T3, exhibiting high stability and significance (*β* = 0.218, SD = 0.018, *t* = 12.012, *p* < 0.001; 95% CI [0.182, 0.253]); life satisfaction at T1 → T2 → T3 (*β* = 0.113, SD = 0.016, *t* = 6.959, *p* < 0.001; 95% CI [0.084, 0.147]).

Because the present study analyzes the same cohort and instruments as Li et al. (2026), the autoregressive estimates for identity awareness and life satisfaction are identical (identity awareness) or nearly identical (life satisfaction) to those reported in that study and should not be regarded as independent findings.

Regarding cross‐lagged effects, the peer relationships at T1 → peer relationships at T2 → physical health at T3 pathway was significant (*β* = 0.043, SD = 0.013, *t* = 3.295, *p* = 0.001; 95% CI [0.018, 0.070]). The identity awareness at T1 → identity awareness at T2 → physical health at T3 path was significant (*β* = 0.035, SD = 0.011, *t* = 3.305, *p* = 0.001; 95% CI [0.015, 0.057]). The peer relationships at T1 → physical health at T2 → physical health at T3 pathway was significant (*β* = 0.050, SD = 0.013, *t* = 3.914, *p* < 0.001; 95% CI [0.025, 0.075]). Regarding the effects on life satisfaction, the peer relationships at T1 → life satisfaction at T2 → life satisfaction at T3 pathway was significant (*β* = 0.020, SD = 0.009, *t* = 2.061, *p* = 0.039; 95% CI [0.001, 0.038]).

Conversely, several indirect pathways did not reach statistical significance. Particularly, identity awareness at T1 did not significantly influence life satisfaction at T3 through life satisfaction at T2 (*β* = 0.013, SD = 0.009, *t* = 1.436, *p* = 0.151; 95% CI [−0.004, .0030]) or peer relationships at T2 (*β* = 0.004, SD = 0.003, *t* = 1.398, *p* = 0.162; 95% CI [−0.001, 0.010]). The path from identity awareness at T1 through identity awareness at T2 to life satisfaction at T3 was also not significant (*β* = 0.010, SD = 0.011, *t* = 0.941, *p* = 0.347; 95% CI [−0.011, 0.032]). Thus, while several exploratory cross‐lagged indirect paths were non‐significant, the theory‐driven longitudinal mediation model provided evidence for specific indirect pathways linking identity awareness to later life satisfaction.

Additionally, identity awareness at T1 did not significantly predict peer relationships at T3 via peer relationships at T2 (*β* = 0.021, SD = 0.013, *t* = 1.623, *p* = 0.105), nor did it affect physical health at T3 via physical health at T2 (*β* = 0.016, SD = 0.011, *t* = 1.541, *p* = 0.123). Other nonsignificant paths included identity awareness at T1 → peer relationships at T2 → physical health at T3 (*β* = 0.004, *p* = 0.153), identity awareness at T1 → identity awareness at T2 → peer relationships at T3 (*β* = 0.017, *p* = 0.136), and identity awareness at T1 → physical health at T2 → life satisfaction at T3 (*β* = 0.003, *p* = 0.221). Notably, the path from physical health at T1 through life satisfaction at T2 to life satisfaction at T3 was also not statistically significant (*β* = 0.006, *p* = 0.558). Thus, from Grades 4 to 6, identity awareness may not exert a consistent/robust indirect effect on subsequent life satisfaction, peer relationships, or health‐related outcomes through intermediate variables.

The findings empirically demonstrated that the autoregressive and some cross‐lagged paths among the variables were stable over time and mutually influential. Notably, the stability of identity awareness and peer relationships, and their indirect effects on physical health perceptions and life satisfaction, were significant (Figure 1).

**FIGURE 1:**
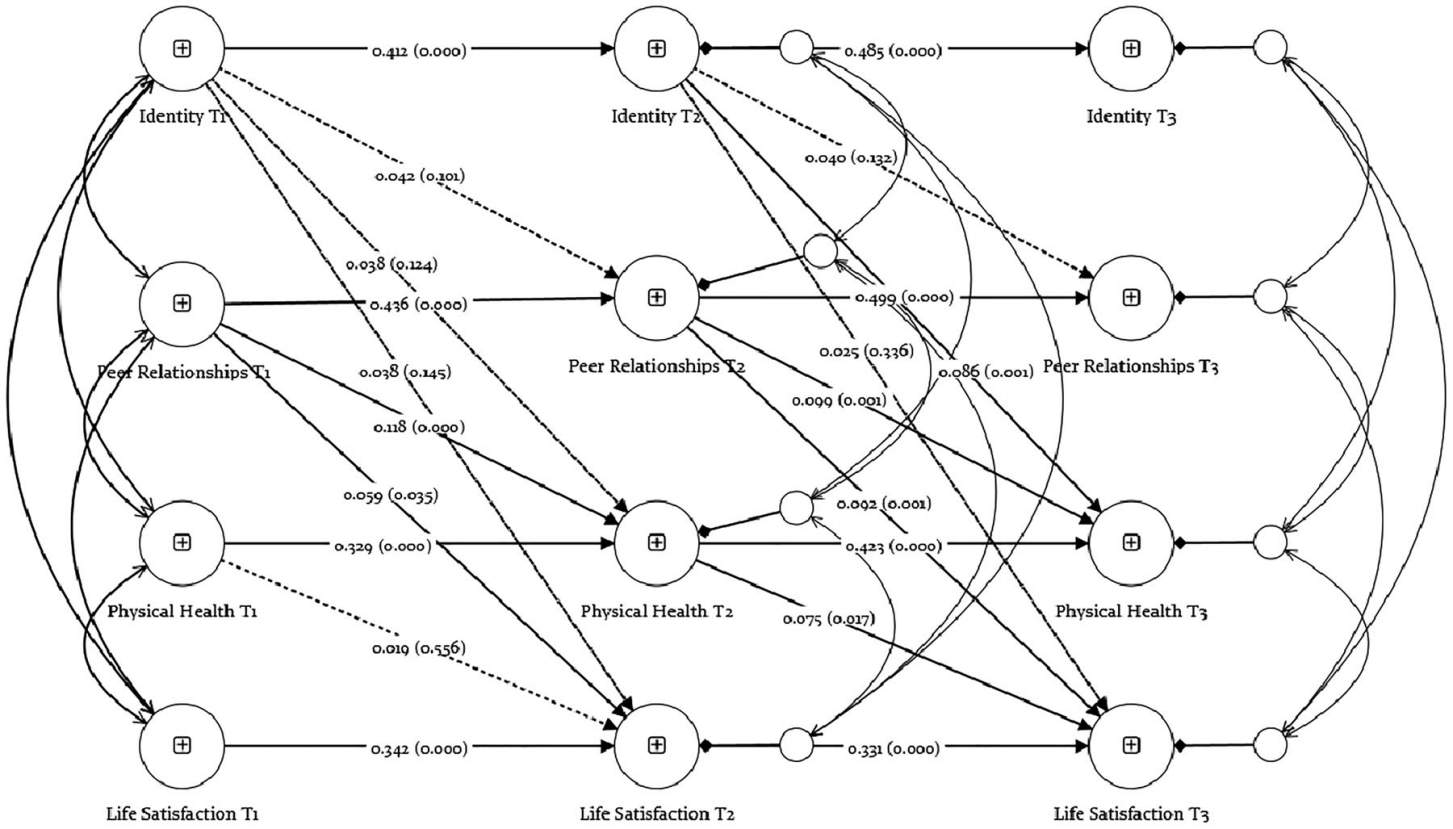
*A*utoregressive cross‐lagged panel model.

#### 3.1.3 Longitudinal Mediating Effects of Identity Awareness, Peer Relationships, Physical Health Perceptions, and Life Satisfaction

The model that was used to examine longitudinal mediation effects exhibited good fit indices (*χ* ^2^ = 218.927, df = 111; CMIN/df = 1.972, GFI = 0.990, AGFI = 0.983, CFI = 0.992, NFI = 0.984, IFI = 0.992, TLI = 0.988, RMSEA = 0.021, and SRMR = 0.023). These analyses adjusted for age, sex, number of close friends, desired educational attainment, and current concerns (i.e., academic performance, school life, and future academic or career planning).

The results indicated that the longitudinal pathway from identity awareness at T1 to life satisfaction at T3 through peer relationships at T2 was significant (*β* = 0.015, SD = 0.005, *t* = 3.184, *p* = 0.001, 95% CI [0.004, 0.024]), implying that a higher identity awareness at T1 positively influenced peer relationships, which indirectly contributed to increased life satisfaction at T3. Additionally, the pathway from identity awareness at T1 to life satisfaction at T3 via physical health at T2 was statistically significant (*β* = 0.013, SD = 0.005, *t* = 2.604, *p* = 0.009, 95% CI [0.007, 0.025]); thus, early identity awareness indirectly enhanced life satisfaction by promoting positive physical health perceptions. Finally, the sequential mediation path from identity awareness at T1 to peer relationships at T2, physical health evaluation at T2, and life satisfaction at T3 was significant (*β* = 0.010, SD = 0.002, *t* = 4.061, *p* < 0.001, 95% CI [0.006, 0.015]). This demonstrated the existence of a chained mediation mechanism in which identity awareness enhanced peer relationships, thereby improving physical health perceptions and increasing life satisfaction (Table 2 and Figure 2).

**TABLE 2:** Longitudinal mediation effect.

Path	*β*	SD	*t*	*p*	95% confidence interval	
					Lower	Upper
Identity awareness T1→physical health perceptions T2→life satisfaction T3	0.013	0.005	2.604	0.009	0.004	0.024
Identity awareness T1→peer relationships T2→life satisfaction T3	0.015	0.005	3.184	0.001	0.007	0.025
Identity awareness T1→peer relationships T2→physical health perceptions T2→life satisfaction T3	0.010	0.002	4.061	< 0.001	0.006	0.015

^*^ *p* < 0.05; ^**^ *p* < 0.01.

**FIGURE 2:**
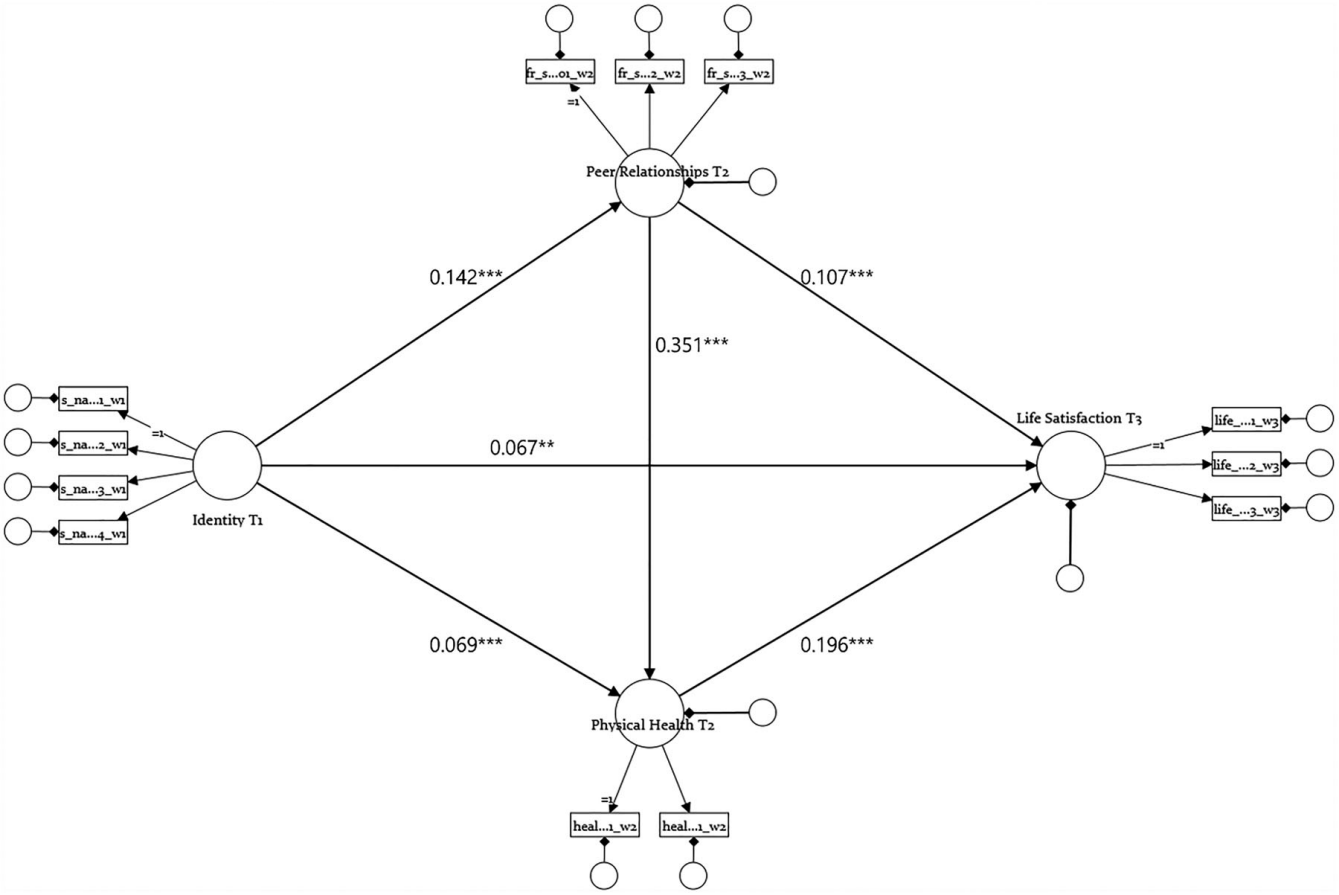
Longitudinal mediation path results. Values on the paths represent direct standardized *β* coefficients. ^***^ *p* < 0.001; ^**^ *p* < 0.01; ^*^ *p* < 0.05.

#### 3.1.4 Moderating Role of PFE in the Relationships Among Identity Awareness, Peer Relationships, Physical Health Perceptions, and Life Satisfaction

To ensure compliance with best practices for moderated mediation analysis, minimize multicollinearity, and ensure interpretive clarity, all continuous variables were mean‐centered before the analysis (West et al. 1996). A PLS‐SEM approach was used, allowing the conditional indirect effect of a predictor on an outcome via a mediator to vary as a function of a moderating variable (Hayes 2018).

The conditional indirect effect of identity awareness on life satisfaction, mediated by physical health perceptions, was significantly moderated by PFE (*β* = 0.080, SE = 0.036, 95% CI [0.009, 0.152]). Therefore, as adolescents perceived their family economic status more positively, the indirect positive effect of identity awareness on life satisfaction via enhanced physical health perceptions became stronger. The model fit indices supported the analysis's adequacy (*R* ^2^ = 0.202; *f* ^2^ = 0.127 for the moderator and 0.124 for the interaction term; *Q* ^2^ = 0.017), indicating the model's sufficient explanatory and predictive power (Table 3).

**TABLE 3:** Moderated mediation effects.

Conditional indirect effect	Path	Index (*β*)	SE	95% confidence interval	
				Lower	Upper
PFE	Identity awareness T1→physical health perceptions T2→life satisfaction T3	0.080	0.036	0.009	0.152

*Note*: Model Fit: *R* ^2^ = 0.202, *f* ^2^ = 0.127 (moderator variable), 0.124 (interaction term), *Q* ^2^ = 0.017.

Abbreviation: PFE, perceived family economic status.

^*^ *p* < 0.05; ^**^ *p* < 0.01.

Additionally, the findings shown in Figure 3 suggested that PFE functioned as a moderator in the life relationship–physical health perceptions relationship. Particularly, as physical health perceptions increased, life satisfaction improved, and this association varied by PFE level. Among adolescents with higher PFE levels (dashed line, Figure 3), physical health perception improvements were associated with steeper increases in life satisfaction. Conversely, for those with lower PFE levels (solid line, Figure 3), the impact of physical health perceptions on life satisfaction was more modest. Therefore, adolescents who perceived their family as economically stable were more likely to link their positive physical health perceptions with their overall life satisfaction. Thus, the beneficial effects of health resources may be amplified or diminished depending on an individual's perceived socioeconomic status.

**FIGURE 3:**
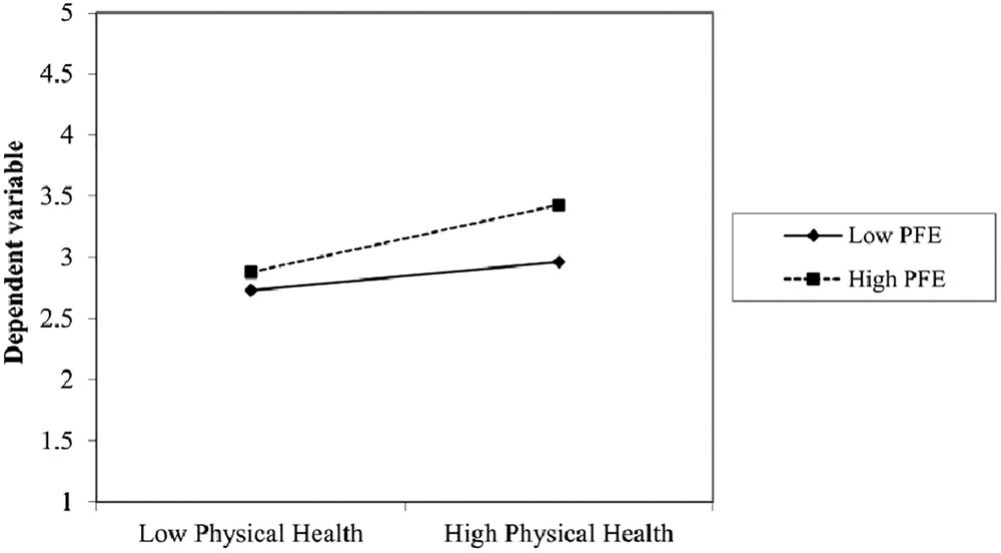
Moderating effect of perceived family economic status. PFE, perceived family economic status.

Figure 4 summarizes the moderated mediation model by combining the standardized path estimates from the longitudinal mediation model with the index of moderated mediation for PFE (index = 0.080, 95% CI [0.009, 0.152]).

**FIGURE 4:**
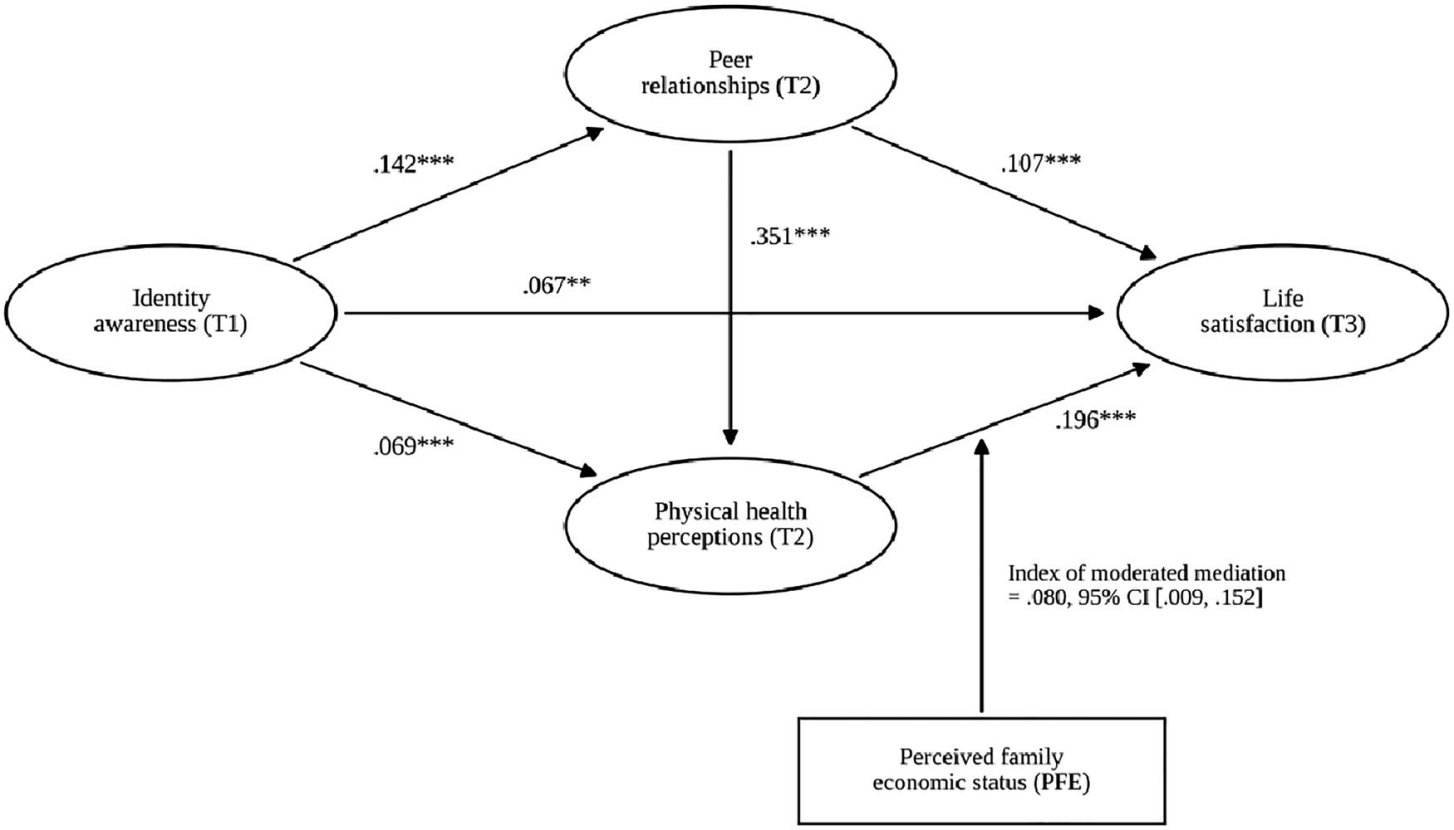
Research model of the moderated mediation with standardized estimates. Path values are standardized coefficients from the longitudinal mediation model (Figure 2). The value on the moderation arrow is the index of moderated mediation from the conditional process analysis (Table 3). PFE, perceived family economic status. ^**^ *p* < 0.01; ^***^ *p* < 0.001.

## 4 Discussion

Establishing a coherent identity across multiple life domains during adolescence is not only a central developmental task but also a critical determinant of overall life satisfaction. This study provides evidence that, among multicultural adolescents, identity awareness is linked to later life satisfaction through peer relationships and physical health perceptions, and that the strength of the health‐related pathway depends on perceived family economic status. Whereas Li et al. (2026) located the identity–life satisfaction link in bicultural acceptance attitudes, the present findings locate it in interpersonal and health resources whose effects are shaped by the family's economic context.

This study makes several significant theoretical and empirical contributions that advance the field of adolescent development and well‐being research. Crucially, it demonstrates that identity awareness has a robust direct effect on life satisfaction and functions indirectly through two clearly delineated, interconnected pathways: peer relationships (psychosocial mechanism) and perceived physical health (somatic mechanism). This dual‐pathway model underscores the complex interplay between internal identity resources and external social and health‐related experiences, emphasizing that adolescent identity is dynamically constructed within multifaceted social environments.

These findings should be read in relation to the research team's earlier analysis of the same cohort (Li et al. 2026). Where the two studies overlap, the results agree: identity awareness and life satisfaction showed substantial autoregressive stability, and identity was associated with later life satisfaction through perceived physical health. Because these overlapping results come from the same adolescents and the same instruments, they represent a consistent pattern within one dataset, not an independent replication. The distinct contribution of the present study lies in two findings that the earlier study did not address. First, it identifies a relational pathway: identity awareness at T1 predicted life satisfaction at T3 through peer relationships at T2 (*β* = 0.015, *p* = 0.001) and through the sequence of peer relationships and physical health perceptions (*β* = 0.010, 95% CI [0.006, 0.015]), whereas the earlier study traced an attitudinal pathway through bicultural acceptance attitudes. Second, it shows that the health‐based pathway is conditional rather than uniform: the indirect effect of identity awareness on life satisfaction via physical health perceptions was moderated by PFE (*β* = 0.080, 95% CI [0.009, 0.152]) and was stronger among adolescents who perceived their family's economic standing more favorably. In the earlier study, the unconditional indirect effect of national identity on life satisfaction via perceived health was only marginally significant (*β* = 0.025, *p* = 0.049, 95% CI [0.000, 0.051]); the present moderation result suggests one possible reason, namely that this pathway may operate more strongly for some adolescents than for others, although this interpretation requires direct testing. Together, the two studies indicate that identity is linked to well‐being among multicultural adolescents through more than one mechanism, attitudinal and relational, and that the health‐based mechanism depends on perceived family economic circumstances.

Furthermore, by incorporating PFE as a moderator, this study broadens the identity–well‐being framework to include critical structural influences. The findings reveal that socioeconomic context is not peripheral but fundamentally shapes how identity awareness translates into well‐being outcomes. Specifically, the positive impact of identity awareness on peer relationships, and consequently, on life satisfaction, is significantly stronger among adolescents who perceive their family's economic status favorably. This highlights PFE as a powerful contextual amplifier of the protective effects of identity, reinforcing the need to consider socioeconomic disparities to understand adolescent adaptation.

Importantly, the use of a rigorous longitudinal moderated mediation design allowed the study to move beyond correlational inference, capturing causal and conditional pathways that reflect the lived realities of multicultural adolescents in a non‐Western context, namely South Korea. This methodological strength positions the study at the forefront of globalizing identity and well‐being research, extending foundational theories beyond Western‐dominated samples and providing culturally sensitive insights.

Grounded in identity theory (Erikson 1968; Marcia 1980), the study confirms that identity awareness drives life satisfaction through mediating social and physical health pathways, with these mechanisms shaped by economic perceptions. The conditional indirect effect emphasizing PFE's moderating role stands out as a critical finding, offering a nuanced understanding of how economic resources or constraints either bolster or limit the benefits of identity development.

By utilizing CB‐SEM and PROCESS‐based PLS‐SEM for robust moderated mediation analysis, this study not only fortifies the empirical evidence for the proposed model but also sets a methodological benchmark for future research. Overall, the results establish identity awareness and its interaction with peer relationships, health perceptions, and socioeconomic context as a pivotal determinant of life satisfaction among multicultural adolescents. The findings highlight urgent directions for interventions aimed at fostering identity coherence and addressing socioeconomic inequalities to enhance adolescent well‐being.

### 4.1 Positive Causal Relationship Between Identity Awareness and Life Satisfaction

First, consistent with our hypotheses, identity awareness at T1 indirectly predicted life satisfaction at T3 via peer relationships at T2 (*β* = 0.015, 95% CI [0.004, 0.024]). This pattern suggests that adolescents who feel a stronger sense of belonging to Korean society are more likely to experience supportive and understanding peer relations 1 year later, which subsequently contributes to higher life satisfaction.

Second, identity awareness at T1 also showed a significant indirect association with life satisfaction at T3 via physical health perceptions at T2 (*β* = 0.013, 95% CI [0.007, 0.025]). In other words, stronger national social identification was associated with more positive perceived physical health at the intermediate wave, which in turn related to greater life satisfaction at the subsequent wave (Becht et al. 2017).

Third, importantly, the sequential pathway identity awareness (T1) → peer relationships (T2) → physical health perceptions (T2) → life satisfaction (T3)—was significant (*β* = 0.010, 95% CI [0.006, 0.015]). This chained mechanism highlights that identity‐related belonging may facilitate social integration, which is linked to more positive health appraisals, and ultimately to greater life satisfaction over time.

The direct path from identity awareness (T1) to life satisfaction (T3) was also significant, indicating both direct and indirect influences. Adolescents with higher identity awareness constructed meaning and value, leading to life satisfaction. All paths remained significant after adjusting for covariates, confirming each pathway's unique explanatory power. The mediating effects of peer relationships and health perceptions were independent and sequential, reinforcing that life satisfaction results from multiple psychosocial mechanisms stemming from identity awareness.

These findings bridge self‐determination theory (Deci and Ryan 2000) and the ecological systems perspective (Bronfenbrenner 2005). Identity awareness provides the psychological basis for adolescents to autonomously manage relationships and accept their bodies, particularly in multicultural settings. Such awareness also protects against cultural tension and social discrimination. This study thus offers a theoretical foundation for understanding how identity–life satisfaction links unfold through practical behavioral pathways. In summary, this study validated three longitudinal pathways linking identity awareness to life satisfaction via peer relationships and health perceptions. The sequential mediation pathway showed the strongest explanatory power. These findings confirm that multicultural adolescents’ life satisfaction depends on the interplay of multiple psychosocial processes over time.

### 4.2 Psychosocial Adjustments Among Multicultural Adolescents: Longitudinal Role of Identity Awareness, Physical Health Perceptions, and PFE

The results showed that family economic status moderated the link between physical health perceptions and life satisfaction. As health perception improved, so did life satisfaction, but more steeply among adolescents who perceived greater economic status (dashed line, Figure 3). For those with lower PFE, even strong health perceptions may not lead to the same gains in satisfaction. Conversely, adolescents who perceive their economic situation as more unstable may have weakened psychosocial pathways linking physical health to life satisfaction, even if their physical health is high.

Self‐determination theory (Deci and Ryan 2000), which emphasizes the interaction between psychosocial resources and environmental perceptions, helps explain this effect. High PFE enhances the link between physical health and basic psychological needs (e.g., autonomy). Low PFE may hinder this link due to social comparison or internal inhibition.

In the PLS‐SEM moderated mediation analysis, the mediating path's strength varied by PFE. The model exhibited both explanatory power and predictive validity. Thus, interventions to improve adolescent well‐being should consider perceived family economic status as a key psychosocial factor.

### 4.3 Practical Implications

First, the confirmation that identity awareness significantly influences life satisfaction and that this relationship is further strengthened through peer relationships and physical health perceptions highlights the need to develop programs that promote identity awareness in schools and community settings. Identity development education or counseling programs that help adolescents explore their values and strengths and recognize themselves within relationships can enhance their long‐term life satisfaction.

Second, the finding that the mediating effect of physical health perceptions is moderated by perceived family economic status suggests that health promotion interventions for multicultural adolescents from vulnerable backgrounds are a vital strategy for supporting identity development and improving life satisfaction. Interventions that encourage healthy lifestyle habits and foster positive body perceptions can serve as indirect pathways for enhancing life satisfaction.(Maeng et al. 2021).

Third, the confirmation that PFE moderates the relationship between physical health perceptions and life satisfaction underscores the importance of class‐sensitive interventions based on adolescents’ socioeconomic perceptions. Practical strategies should be tailored to reflect the interaction between psychological factors (e.g., identity) and situational factors (e.g., perceived family environment).

Fourth, improving multicultural adolescents’ life satisfaction requires an integrated approach, not one based solely on isolated psychological factors. Interventions should address identity, peer relationships, physical perceptions, and family context together. A multidimensional support system that integrates counseling, health education, and peer relationships in school settings can significantly enhance the social adaptation and overall well‐being of multicultural adolescents. Programs, policies, and interventions should be designed with consideration of how identity formation, health, interpersonal relationships, and socioeconomic perceptions intersect. Such holistic, context‐sensitive efforts may yield far‐reaching results in promoting multicultural adolescents’ psychological and social development.

Fifth, by applying an autoregressive cross‐lagged panel model, this study empirically confirmed that the causal relationship between identity awareness and life satisfaction unfolds as a cyclical, strengthening process over time, rather than as a one‐time effect. This analytical approach offers a dynamic and novel understanding of psychosocial change during adolescence, emphasizing the importance of well‐timed, developmentally sensitive interventions in real‐world settings.

### 4.4 Limitations and Future Research

This study has several limitations. First, identity awareness was measured using self‐reported questionnaires; however, identity is a construct with fluid characteristics. Therefore, future studies should adopt a multi‐method approach (e.g., peer/parent reports on identity or behavioral observation) to assess identity awareness (Eid and Diener 2006; Kosinski et al. 2013). Additionally, time series modeling based on repeated measures designs or using mobile‐based ecological momentary assessment would also be appropriate (Becht et al. 2017).

Second, our analyses were primarily variable‐centered. Identity development in multicultural contexts may unfold via heterogeneous subgroups (e.g., stable‐high, increasing, or chronically low identification) and through multidimensional configurations of ethnicities and identities. Future studies should therefore apply person‐centered approaches (e.g., latent profile/transition or growth mixture models) to capture distinct identity trajectories and test whether the proposed pathways differ across these subgroups (Becht et al. 2017).

Third, this study utilized an autoregressive cross‐lagged panel model to analyze the longitudinal stability of variables and their causal pathways. Future studies should introduce a random intercept cross‐lagged panel model (Hamaker et al. 2015) to distinguish between within‐ and between‐person effects.

## 5 Conclusions

This study advances understanding of how identity awareness contributes to adolescents’ life satisfaction by proposing and validating a longitudinal, sequential mediation model. By demonstrating that peer relationships and perceived physical health function as key mediators, the study offers a nuanced, temporally grounded account of the identity–life satisfaction link. This approach moves beyond cross‐sectional snapshots and highlights the developmental nature of these processes over time.

Read alongside the research team's earlier analysis of the same cohort (Li et al. 2026), which identified a pathway through bicultural acceptance attitudes, the present study adds two findings: a relational pathway through peer relationships, and the conditioning of the health‐based pathway by perceived family economic status. These two findings constitute the distinct contribution of the present analysis.

By focusing on multicultural adolescents in South Korea, the study addresses a critical gap by contextualizing identity development within a collectivistic, academically competitive environment. It broadens the theoretical scope of identity research and shows how cultural norms and social interactions shape identity processes.

The study makes a distinctive contribution by integrating social, cultural, and health dimensions into the study of life satisfaction. It reinforces the importance of identity clarity among adolescents and introduces a dynamic, culturally embedded framework to guide future research and interventions. Through its longitudinal design and culturally sensitive lens, the study offers a robust model for understanding how identity fosters life satisfaction among adolescents navigating complex developmental contexts.

## Author Contributions

**Suyeon Lee**: supervision, validation, writing – review and editing. **Yiran Li**: conceptualization, investigation, funding acquisition, writing – original draft, methodology, visualization, validation, writing – review and editing, project administration, formal analysis, software, data curation, supervision, resources.

## Funding

The authors have nothing to report.

## Data Availability

The data that support the findings of this study are available on request from the corresponding author. The data are not publicly available due to privacy or ethical restrictions.

## References

[brb371317-bib-0002] Aral, T. , L. P. Juang , M. Schwarzenthal , M. K. Schachner , and B. G. Adams . 2025. “The Social Contexts of Cultural Identity Among Adolescents of Immigrant Descent: Separate and Interacting Relations of Family and School Ethnic‐Racial Socialization.” Journal of Early Adolescence 45, no. 4: 421–450. 10.1177/02724316241251401.

[brb371317-bib-0003] Arnett, J. J. 2002. “The Psychology of Globalization.” American Psychologist 57, no. 10: 774–783. 10.1037/0003-066X.57.10.774. 12369500

[brb371317-bib-0004] Arnett Jensen, L. 2003. “Coming of Age in a Multicultural World: Globalization and Adolescent Cultural Identity Formation.” Applied Developmental Science 7, no. 3: 189–196. 10.1207/S1532480XADS0703_10.

[brb371317-bib-0005] Bagci, S. C. , and A. Rutland . 2020. “Ethnic Majority and Minority Youth in Multicultural Societies.” In Youth in Superdiverse Societies, edited by P. F. Titzmann and P. Jugert , 177–195. Routledge.

[brb371317-bib-0006] Becht, A. I. , S. A. Nelemans , S. J. Branje , W. A. Vollebergh , H. M. Koot , and W. H. Meeus . 2017. “Identity Uncertainty and Commitment Making Across Adolescence: Five‐Year Within‐Person Associations Using Daily Identity Reports.” Developmental Psychology 53, no. 11: 2103–2112. 10.1037/dev0000374. 29094973

[brb371317-bib-0007] Berry, J. W. 1997. “Immigration, Acculturation, and Adaptation.” Applied Psychology 46, no. 1: 5–34.

[brb371317-bib-0008] Berry, J. W. 2003. “Conceptual approaches to acculturation.” Acculturation: Advances in theory, measurement, and applied research, edited by P. Balls Organista K. M. Chun , and G. Marín , 17–37. American Psychological Association. 10.1037/10472-004.

[brb371317-bib-0009] Branje, S. J. , M. van Doorn , I. van der Valk , and W. Meeus . 2009. “Parent–Adolescent Conflicts, Conflict Resolution Types, and Adolescent Adjustment.” Journal of Applied Developmental Psychology 30, no. 2: 195–204. 10.1016/j.appdev.2008.12.004.

[brb371317-bib-0010] Bronfenbrenner, U. 2005. “Ecological Systems Theory (1992).” In Making Human Beings Human: Bioecological Perspectives on Human Development, edited by U. Bronfenbrenner , 106–173.Sage Publications Ltd.

[brb371317-bib-0011] Byrne, B. M. 2012. A Primer of LISREL: Basic Applications and Programming for Confirmatory Factor Analytic Models. Springer Science & Business Media.

[brb371317-bib-0012] Cawte, J. 1972. Cruel, Poor and Brutal Nations: The Assessment of Mental Health in an Australian Aboriginal Community by Short‐Stay Psychiatric Field Team Methods . University of Hawaii Press.

[brb371317-bib-0013] Cham, H. , E. Reshetnyak , B. Rosenfeld , and W. Breitbart . 2017. “Full Information Maximum Likelihood Estimation for Latent Variable Interactions With Incomplete Indicators.” Multivariate Behavioral Research 52, no. 1: 12–30. 10.1080/00273171.2016.1245600. PMC5489914 27834491

[brb371317-bib-0014] Chen, E. , and G. E. Miller . 2013. “Socioeconomic Status and Health: Mediating and Moderating Factors.” Annual Review of Clinical Psychology 9, no. 1: 723–749. 10.1146/annurev-clinpsy-050212-185634. 23245339

[brb371317-bib-0015] Chen, F. F. 2007. “Sensitivity of Goodness of Fit Indexes to Lack of Measurement Invariance.” Structural Equation Modeling: A Multidisciplinary Journal 14, no. 3: 464–504. 10.1080/10705510701301834.

[brb371317-bib-0017] Cho, Y. , and H. Jeong . 2024. “The Longitudinal Mediating Effect of Bicultural Acceptance Attitudes on the Relationship Between Cultural Adaptation Stress and National Identity Among Multicultural Adolescents.” Korean Journal of Youth Studies 35, no. 4: 231–253. 10.14816/sky.2024.35.4.231.

[brb371317-bib-0018] Cohen, S. , and T. A. Wills . 1985. “Stress, Social Support, and the Buffering Hypothesis.” Psychological Bulletin 98, no. 2: 310–357. 10.1037/0033-2909.98.2.310. 3901065

[brb371317-bib-0020] Conger, R. D. , K. J. Conger , G. H. Elder Jr , F. O. Lorenz , R. L. Simons , and L. B. Whitbeck . 1992. “A Family Process Model of Economic Hardship and Adjustment of Early Adolescent Boys.” Child Development 63, no. 3: 526. 10.2307/1131344. 1600820

[brb371317-bib-0021] Conger, R. D. , and M. B. Donnellan . 2007. “An Interactionist Perspective on the Socioeconomic Context of Human Development.” Annual Review of Psychology 58, no. 1: 175–199. 10.1146/annurev.psych.58.110405.085551. 16903807

[brb371317-bib-0023] Crocetti, E. , T. A. Klimstra , L. Keijsers , W. W. Hale , and W. Meeus . 2009. “Anxiety Trajectories and Identity Development in Adolescence: A Five‐Wave Longitudinal Study.” Journal of Youth and Adolescence 38, no. 6: 839–849. 10.1007/s10964-008-9350-y. 19636785

[brb371317-bib-0023a] Crocetti, E. 2017. “Identity Formation in Adolescence: The Dynamic of Forming and Consolidating Identity Commitments.” Child Development Perspectives 11, no. 2: 145–150. 10.1111/cdep.12226.

[brb371317-bib-0024] Crocetti, E. , M. Rubini , and S. Mastrotheodoros . 2023. “Identity Development in Multicultural Societies: An Integrative Perspective.” Current Opinion in Psychology 48: 101537. 10.1016/j.copsyc.2022.101537.

[brb371317-bib-0024a] Crocetti, E. , S. Karataş , S. Branje , B. Bobba , and M. Rubini . 2024. “Navigating across heritage and destination cultures: How personal identity and social identification processes relate to domain-specific acculturation orientations in adolescence.” Journal of Youth and Adolescence 53, no. 2: 397415. 10.1007/s10964-023-01870-y. PMC10764387 37775692

[brb371317-bib-0025] Deci, E. L. , and R. M. Ryan . 2000. “The "What" and "Why" of Goal Pursuits: Human Needs and the Self‐Determination of Behavior.” Psychological Inquiry 11, no. 4: 227–268. 10.1207/S15327965PLI1104_01.

[brb371317-bib-0026] De Lise, F. , K. Luyckx , and E. Crocetti . 2024. “Identity Matters for Well‐Being: The Longitudinal Associations Between Identity Processes and Well‐Being in Adolescents With Different Cultural Backgrounds.” Journal of Youth and Adolescence 53, no. 4: 910–926. 10.1007/s10964-023-01901-8 PMC10879306 37938483

[brb371317-bib-0027] Diener, E. , E. M. Suh , R. E. Lucas , and H. L. Smith . 1999. “Subjective Well‐Being: Three Decades of Progress.” Psychological Bulletin 125, no. 2: 276–302. 10.1037/0033-2909.125.2.276.

[brb371317-bib-0028] Eid, M. E. , and E. E. Diener . 2006. Handbook of Multimethod Measurement in Psychology. American Psychological Association. 10.1037/11383-000.

[brb371317-bib-0029] Erikson, E. H. 1968. Identity, Youth and Crisis. W. W. Norton & Company.

[brb371317-bib-0030] Evans, G. W. , and P. Kim . 2013. “Childhood Poverty, Chronic Stress, Self‐Regulation, and Coping.” Child Development Perspectives 7, no. 1: 43–48. 10.1111/cdep.12013.

[brb371317-bib-0031] Gallo, L. C. , and K. A. Matthews . 2003. “Understanding the Association Between Socioeconomic Status and Physical Health: Do Negative Emotions Play a Role?.” Psychological Bulletin 129, no. 1: 10–51. 10.1037/0033-2909.129.1.10. 12555793

[brb371317-bib-0032] Gardiner, H. W. , and C. Kosmitzki . 2018. Lives Across Cultures: Cross‐Cultural Human Development. 6th ed. Pearson.

[brb371317-bib-0033] Goodman, E. , N. E. Adler , I. Kawachi , A. L. Frazier , B. Huang , and G. A. Colditz . 2001. “Adolescents' Perceptions of Social Status: Development and Evaluation of a New Indicator.” Pediatrics 108, no. 2: e31–e31. 10.1542/peds.108.2.e31. 11483841

[brb371317-bib-0034] Goodman, E. , B. Huang , T. Schafer‐Kalkhoff , and N. E. Adler . 2007. “Perceived Socioeconomic Status: A New Type of Identity That Influences Adolescents' Self‐Rated Health.” Journal of Adolescent Health 41, no. 5: 479–487. 10.1016/j.jadohealth.2007.05.020. PMC2204090 17950168

[brb371317-bib-0035] Hair, J. F. , M. Sarstedt , C. M. Ringle , and S. P. Gudergan . 2024. Advanced Issues in Partial Least Squares Structural Equation Modeling (PLS‐SEM) (2e). Sage.

[brb371317-bib-0036] Hamaker, E. L. , R. M. Kuiper , and R. P. Grasman . 2015. “A Critique of the Cross‐Lagged Panel Model.” Psychological Methods 20, no. 1: 102–116. 10.1037/a0038889. 25822208

[brb371317-bib-0037] Hatano, K. , K. Sugimura , and E. Crocetti . 2016. “Looking at the Dark and Bright Sides of Identity Formation: New Insights From Adolescents and Emerging Adults in Japan.” Journal of Adolescence 47: 156–168. 10.1016/j.adolescence.2015.09.008. 26522882

[brb371317-bib-0038] Hayes, A. F. 2018. Introduction to Mediation, Moderation, and Conditional Process Analysis: A Regression‐Based Approach. 2nd ed. Guildford Press.

[brb371317-bib-0039] Hayes, A. F. , A. K. Montoya , and N. J. Rockwood . 2017. “The Analysis of Mechanisms and Their Contingencies: PROCESS Versus Structural Equation Modeling.” Australasian Marketing Journal 25, no. 1: 76–81. 10.1016/j.ausmj.2017.02.001.

[brb371317-bib-0040] Hong, Y. Y. , S. Zhan , M. W. Morris , and V. Benet‐Martínez . 2016. “Multicultural Identity Processes.” Current Opinion in Psychology 8: 49–53. 10.1016/j.copsyc.2015.09.020. 29506802

[brb371317-bib-0041] Hwang, J.‐g. 1997. “Life Satisfaction, Psychological Health, and Physical Health Among Korean Adolescents: Psychological and Social factors.” In Korean Youth Culture: Psychological and Social Factors (Research Papers 97–8), edited by Education and Ethics Research Institute, 291–343. Korean Institute of Mental Culture.

[brb371317-bib-0042] Hwang, Y.‐J. , and K.‐K. Kim . 2006. “Determinants of Student's Satisfaction With General High School Students.” Korean Journal of Sociology of Education 16, no. 2: 181–203.

[brb371317-bib-0043] Jeong, D. , and J. Paik . 2020. “The Effects of MultiCultural Adolescent's Ego‐Resilience, Biculture‐Acceptance, Bullying and Parenting Attitude on Their Intention of School Dropout.” Journal of Convergence for Information Technology 10, no. 10: 161–173. 10.22156/CS4SMB.2020.10.10.161.

[brb371317-bib-0044] Kang, M. , A. Min , and H. Min . 2022. “Predicting Multicultural Adolescents' Depression in South Korea: Roles of Ego‐Resilience and Social Support.” Journal of Pediatric Nursing 65: e93–e98. 10.1016/j.pedn.2022.03.012. 35422366

[brb371317-bib-0045] Kang, Y. 2019. The Relationship Between Cultural Adaptation Stress and Bicultural Acceptance Attitudes Among Adolescents From Multicultural Families: Developmental Trajectories and Developmental Outcomes . Doctoral dissertation, Catholic University of Korea.

[brb371317-bib-0046] Karaś, D. , and J. Cieciuch . 2018. “The Relationship Between Identity Processes and Well‐Being in Various Life Domains.” Personality and Individual Differences 121: 111–119.

[brb371317-bib-0047] Karaś, D. , J. Cieciuch , and C. L. Keyes . 2014. “The Polish Adaptation of the Mental Health Continuum‐short Form (MHC‐SF).” Personality and Individual Differences 69: 104–109.

[brb371317-bib-0048] Karataş, S. , E. Crocetti , S. J. Schwartz , and M. Rubini . 2023. “Developmental Trajectories of Ethnic and National Identities in Adolescents From Migrant Families: The Role of Social Identification With family and Classmates.” European Journal of Personality 37, no. 6: 705–722. 10.1177/08902070221149602.

[brb371317-bib-0049] Keyes, C. L. M. 2005. “Mental Illness and/or Mental Health? Investigating Axioms of the Complete state Model of Health.” Journal of Consulting and Clinical Psychology 73, no. 3: 539–548. 10.1037/0022-006X.73.3.539. 15982151

[brb371317-bib-0051] Kim, J.‐N. 2013. “Development and Validation of a Shortened Version of the School Adaptation Scale for Middle and High School Students.” Journal of the Korean Psychological Association: Counseling and Psychotherapy 25, no. 3: 497–517.

[brb371317-bib-0052] Kim, J. K. , H. J. Baek , H. J. Lim , and G. O. Lee . 2010. Korean Children and Youth Panel Survey 2010 , Vol. I (Research Report 10‐R01). National Youth Policy Institute.

[brb371317-bib-0053] Kim, S. M. , and H. O. Kim . 2022. “Effect of Acculturative Stress on Multicultural Adolescents' Life Satisfaction: Sequential Multiple Mediating Effects of Bicultural Acceptance Attitude, Self‐Esteem, and Social Withdrawal—Using the 2016 Multicultural Adolescents Panel Study.” Journal of Korean Academy of Nursing 52, no. 3: 324–340. 10.4040/jkan.22030. 35818880

[brb371317-bib-0054] Kim, S. Y. , J. Y. Lim , S. W. Kim , et al. 2006. Korean Youth Development Index Survey: Validation of Outcome Measurement Indicators (Research Report 06‐R02) . Korea Institute for Youth Development.

[brb371317-bib-0056] Kline, R. B. 2023. Principles and Practice of Structural Equation Modeling, 5th ed. Guilford Press.

[brb371317-bib-0057] Kosinski, M. , D. Stillwell , and T. Graepel . 2013. “Private Traits and Attributes are Predictable From Digital Records of Human Behavior.” Proceedings of the National Academy of Sciences 110, no. 15: 5802–5805. 10.1073/pnas.1218772110. PMC3625324 23479631

[brb371317-bib-0058] KOSIS National Statistics Portal . 2023. National Survey on the Status of Multicultural Families. Statistics Korea.

[brb371317-bib-0059] Li, Y. , Y. Gu , and I.‐J. Yoon . 2026. “National Identity and Life Satisfaction Among Multicultural Adolescents in South Korea: The Sequential Mediating Roles of Bicultural Attitudes and Perceived Health.” Journal of Cross‐Cultural Psychology 57, no. 4: 675–698. 10.1177/00220221251412881.

[brb371317-bib-0060] Maeng, M. S. , M. J. Chon , and Y. S. Joung . 2021. “The Effect of Multicultural Adolescents' body Satisfaction on Life Satisfaction: The Mediating Effect of Depreesion [sic] and Friendship.” Journal of Industrial Convergence 19, no. 4: 127–134. 10.22678/JIC.2021.19.4.127.

[brb371317-bib-0061] Marcia, J. 1980. “Identity in Adolescence.” In Handbook of Adolescent Psychology, edited by J. Adelson , 159–187. Wiley.

[brb371317-bib-0062] Markus, H. R. , and S. Kitayama . 1991. “Cultural Variation in the Self‐Concept.” In The Self: Interdisciplinary Approaches, edited by G. R. Goethals and J. Strauss, 18–48. Springer. 10.1007/978-1-4684-8264-510.1007/978-1-4684-8264-5_2.

[brb371317-bib-0063] Masarik, A. S. , and R. D. Conger . 2017. “Stress and Child Development: A Review of the Family Stress Model.” Current Opinion in Psychology 13: 85–90. 10.1016/j.copsyc.2016.05.008. 28813301

[brb371317-bib-0064] Mastrotheodoros, S. , J. Hillekens , M. Miklikowska , B. E. Palladino , and F. Lionetti . 2024. “Family Functioning, Identity Commitments, and School Value Among Ethnic Minority and Ethnic Majority Adolescents.” Journal of Youth and Adolescence 53, no. 6: 1323–1340. 10.1007/s10964-024-01972-1. PMC11045604 38553579

[brb371317-bib-0066] Mercer, N. , E. Crocetti , S. Branje , P. van Lier , and W. Meeus . 2017. “Linking Delinquency and Personal Identity Formation Across Adolescence: Examining Between‐ and Within‐person Associations.” Developmental Psychology 53, no. 11: 2182–2194. 10.1037/dev0000351. 29094979

[brb371317-bib-0068] Miller, R. L. , and T. Collette . 2019. “Multicultural Identity Development: Theory and Research.” In Cross‐Cultural Psychology: Contemporary Themes and Perspectives, edited by K. D. Keith , 2nd Ed., 614–631. Wiley‐Blackwell. 10.1002/9781119519348.

[brb371317-bib-0069] Ministry of Education, & Korea Educational Development Institute . 2018. Internal Data From the KEDI Center for Educational Statistics [Unpublished internal data set].

[brb371317-bib-0070] Mok, A. 2022. “Feeling at Home in Two Cultural Worlds: Bicultural Identity Integration Moderates Felt Authenticity.” Journal of Cross‐Cultural Psychology 53, no. 2: 179–212. 10.1177/00220221211072798.

[brb371317-bib-0071] Moon, S. H. , M. K. Lee , M. J. Lee , H. J. Shim , and H. H. Cho . 2018. “Factors Influencing the Experience of Depression Among School‐Aged Children From Multicultural Families.” Child Health Nursing Research 24, no. 4: 434–442. 10.4094/chnr.2018.24.4.434.

[brb371317-bib-0072] Motti‐Stefanidi, F. 2015. “Identity Development in the Context of the Risk and Resilience framework.” In The Oxford Handbook of Identity Development, K. C. McLean , and M. Syed (Eds.), (472–489). Oxford University Press. 10.1093/oxfordhb/9780199936564.013.014.

[brb371317-bib-0073] Statistics Korea . 2023. 2022 Multicultural Population Dynamics Statistics ..[Press release]. https://mods.go.kr/board.es?act=view&bid=204&list_no=428278&mid=a10301010000.

[brb371317-bib-0074] Nguyen, A. M. D. , and V. Benet‐Martínez . 2013. “Biculturalism and Adjustment: A Meta‐analysis.” Journal of Cross‐Cultural Psychology 44, no. 1: 122–159. 10.1177/0022022111435097.

[brb371317-bib-0075] Park, J. 2023. “The Structural Relationship Between Cultural Adaptation Stress and Dual Cultural Acceptance Attitudes of Children From Multicultural Families and National Identity: Focusing on the Experience of Multicultural Family Support Policies.” Multicultural Education Research 16, no. 1: 165–190.

[brb371317-bib-0077] Quon, E. C. , and J. J. McGrath . 2014. “Subjective Socioeconomic Status and Adolescent Health: A Meta‐Analysis.” Health Psychology 33, no. 5: 433–447. 10.1037/a0033716. PMC5756083 24245837

[brb371317-bib-0078] Ratzmann, M. , S. P. Gudergan , and R. Bouncken . 2016. “Capturing Heterogeneity and PLS‐SEM Prediction Ability: Alliance Governance and Innovation.” Journal of Business Research 69, no. 10: 4593–4603. 10.1016/j.jbusres.2016.03.051.

[brb371317-bib-0079] Reifsnider, E. , M. Gallagher , and B. Forgione . 2005. “Using Ecological Models in Research on Health Disparities.” Journal of Professional Nursing 21, no. 4: 216–222. 10.1016/j.profnurs.2005.05.006. 16061168

[brb371317-bib-0080] Romero, A. , B. Piña‐Watson , A. K. Stevens , et al. 2020. “Disentangling Relationships Between Bicultural Stress and Mental Well‐Being Among Latinx Immigrant Adolescents.” Journal of Consulting and Clinical Psychology 88, no. 2: 149–159. 10.1037/ccp0000466. PMC7236092 31894996

[brb371317-bib-0081] Ryan, R. M. , and E. L. Deci . 2001. “On Happiness and Human Potentials: A Review of Research on Hedonic and Eudaimonic Well‐Being.” Annual Review of Psychology 52, no. 1: 141–166. 10.1146/annurev.psych.52.1.141. 11148302

[brb371317-bib-0082] Ryff, C. D. 1989. “Happiness Is Everything, or Is It? Explorations on the Meaning of Psychological Well‐Being.” Journal of Personality and Social Psychology 57, no. 6: 1069–1081. 10.1037/0022-3514.57.6.1069.

[brb371317-bib-0083] Ryff, C. D. 2014. “Self‐Realisation and Meaning Making in the Face of Adversity: A Eudaimonic Approach to Human Resilience.” Journal of Psychology in Africa 24, no. 1: 1–12. 10.1080/14330237.2014.904098. PMC4243302 25435804

[brb371317-bib-0084] Schwartz, S. J. , J. B. Unger , B. L. Zamboanga , and J. Szapocznik . 2010. “Rethinking the Concept of Acculturation: Implications for Theory and Research.” American Psychologist 65, no. 4: 237–251. 10.1037/a0019330. PMC3700543 20455618

[brb371317-bib-0085] Seligman, M. E. P. , and M. Csikszentmihalyi . 2000. “Positive Psychology: An Introduction.” American Psychologist 55, no. 1: 5–14. 10.1037/0003-066X.55.1.5. 11392865

[brb371317-bib-0086] Seong, H.‐K. 2001. “Development of the Korean Version of the Social Identity Scale.” Journal of the Korean Psychological Association: Social and Personality 15, no. 3: 33–48.

[brb371317-bib-0087] Seo, M.‐O. 2022. “The Influence of Interpersonal Skills on Life Satisfaction Among College Students and the Mediating Effect of Five Personality Traits.” Learner‐Centered Curriculum Education Research 22, no. 24: 657–670.

[brb371317-bib-0088] Shen, J. , and I. Kogan . 2021. “Gendered Body Mass and Life Satisfaction Among Youth in Three Western European Immigrant‐Receiving Countries.” Frontiers in Sociology 6: 695374. 10.3389/fsoc.2021.695374. PMC8705931 34957290

[brb371317-bib-0089] West, S. G. , L. S. Aiken , and J. L. Krull . 1996. “Experimental Personality Designs: Analyzing Categorical by Continuous Variable Interactions.” Journal of Personality 64, no. 1: 1–48. 10.1111/j.1467-6494.1996.tb00813.x. 8656311

[brb371317-bib-0090] Wiley, S. , and K. Deaux . 2010. “The Bicultural Identity Performance of Immigrants.” In Identity and Participation in Culturally Diverse Societies: A Multidisciplinary Perspective, edited by A. E. Azzi , X. Chryssochoou , B. Klandermans , and B. Simon , 49–68. Wiley. 10.1002/9781444328158.

[brb371317-bib-0092] Yun, H. J. , and Y. J. Kwon . 2025. “Structural Relationship Analysis of Factors Influencing Life Satisfaction of Multicultural Adolescents: A Comparative Study Based on Ethnic Identity Types.” Multicultural Child and Youth Studies 10, no. 1: 1–34. 10.56023/mcyca.2025.10.1.1.

